# Nurse-Led Functional Nutrition Care Model (FUNCARE): Integrating Biologic and Behavioral Nursing Approaches to Improve Glycemic Outcomes in Type 2 Diabetes

**DOI:** 10.1016/j.cdnut.2026.109555

**Published:** 2026-10-17

**Authors:** Andina Setyawati, Nuurhidayat Jafar, Whika Febria Dewatisari, Putri Cahaya Situmorang

**Affiliations:** 1 Department of Medical-Surgical Nursing, Faculty of Nursing, Universitas Hasanuddin, Makassar, Indonesia; 2 Department of Community Health Nursing, Faculty of Nursing, Universitas Hasanuddin, Makassar, Indonesia; 3 Department of Biology, Faculty of Science and Technology, Universitas Terbuka, South Tangerang, Indonesia; 4 Department of Biology, Faculty of Mathematics and Natural Sciences, Universitas Sumatera Utara, Medan, Indonesia

**Keywords:** type 2 diabetes mellitus, nursing care, functional foods, *Gynura procumbens*, insulin resistance, vascular health

## Abstract

**Background:**

Functional foods may complement nurse-led diabetes care. *Gynura procumbens*, a flavonoid-rich plant with antihyperglycemic and vasoprotective potential, was incorporated into a nurse-led functional nutrition care model (FUNCARE) for adults with type 2 diabetes mellitus (T2DM).

**Objectives:**

To evaluate the glycemic and vascular effects, acceptability, and safety of *G. procumbens*–enriched cookies delivered within FUNCARE.

**Methods:**

In this randomized, placebo-controlled, 4-arm trial, 246 adults with T2DM from 5 community health centers received placebo cookies or cookies containing 5%, 10%, or 20% *G. procumbens* extract. All participants consumed 2 cookies daily for 12 wk and received standardized nurse-led coaching. Outcomes included fasting plasma glucose (FPG), glycated hemoglobin (HbA1c), homeostasis model assessment of insulin resistance (HOMA-IR), ankle-brachial index (ABI), and matrix metalloproteinase-9 (MMP-9)/tissue inhibitor of metalloproteinase-1 (TIMP-1) ratio. Week-12 outcomes were analyzed by analysis of covariance adjusted for baseline values, body mass index, and diabetes duration, with Dunnett-adjusted contrasts compared with placebo.

**Results:**

No significant treatment-group effect was observed for FPG (*P* = 0.61). HbA1c differed significantly across groups (*P* < 0.001); compared with placebo, adjusted HbA1c was lower by 0.59 percentage points in the 5% group [95% confidence interval (CI), −1.01, −0.17; *P* = 0.006), 0.84 in the 10% group (95% CI: −1.26, −0.42; *P* <0.001), and 2.13 in the 20% group (95% CI: −2.56, −1.70; *P* <0.001). HOMA-IR was significantly lower than placebo in the 10% and 20% groups (both *P* < 0.001). Treatment-group effects were also significant for ABI (*P* = 0.004) and MMP-9/TIMP-1 ratio (*P* < 0.001). Adherence exceeded 90%, acceptability was 96%, and no serious adverse events occurred.

**Conclusions:**

: Within standardized nurse-led care, *G. procumbens*–enriched cookies are associated with lower HbA1c and lower HOMA-IR compared with placebo, whereas FPG remains unchanged; selected vascular outcomes also differ. Because all groups receive the same nurse-led coaching, the independent effect of FUNCARE cannot be determined.

This trial was registered at clinicaltrials.gov as NCT07151833.

## Introduction

Type 2 diabetes mellitus (T2DM) is one of the most prevalent chronic diseases worldwide and a major contributor to morbidity and mortality [1]. According to the International Diabetes Federation (2025), ∼537 million adults are living with diabetes globally, and this number is projected to rise to 643 million by 2030 [2]. In Southeast Asia, the prevalence continues to increase rapidly, with Indonesia ranking among the top 10 countries, reporting over 19.5 million adults affected by diabetes [3]. Despite advances in pharmacologic management, nearly half of patients fail to achieve optimal glycemic control, leading to long-term complications such as neuropathy, nephropathy, and peripheral vascular disease [4], [5], [6].

Persistent hyperglycemia and oxidative stress are known to disrupt endothelial integrity and microvascular circulation [7]. These biologic mechanisms highlight the need for complementary interventions that target both metabolic regulation and vascular protection. Functional foods containing bioactive compounds have shown promise as adjuncts to diabetes care. Among them, *Gynura procumbens*, a tropical medicinal plant rich in flavonoids and phenolics, has demonstrated antihyperglycemic, antioxidant, and vasoprotective properties in experimental studies. Its mechanisms include enhancing superoxide dismutase activity, reducing p53-mediated apoptosis, and improving insulin sensitivity [8], [9]. However, clinical evidence supporting these effects in human populations remains scarce, particularly within community-based nursing interventions [10].

From a nursing perspective, addressing diabetes requires an integrative approach that combines biologic insight with behavioral modification [11], [12]. Nurses play a key role in promoting self-care management, dietary adherence, and health coaching. Integrating functional nutrition into nurse-led care can bridge the gap between biologic efficacy and behavioral sustainability, especially in primary care settings [13].

Therefore, this study aimed to evaluate the functional nutrition care model (FUNCARE), a model combining *G. procumbens*–enriched functional cookies (GlucoBites) with structured nurse-led coaching, to improve glycemic control and vascular health among adults with T2DM. This research sought to provide biologic and behavioral evidence for the effectiveness of functional nutrition integrated into nursing practice.

## Methods

### Study design

This study employed a double-blind, randomized controlled trial with a parallel-group design to evaluate the effectiveness of the nurse-led FUNCARE using *G. procumbens*–enriched functional cookies (GlucoBites) on glycemic and vascular outcomes among adults with T2DM. The study followed the CONSORT 2010 guidelines for reporting randomized trials. A completed CONSORT checklist is provided in Supplemental Material 1.

### Setting and participants

The trial was conducted between June and October 2025 across 5 community health centers in 3 regencies in Indonesia. Eligible participants were adults aged 40–65 y with a clinical diagnosis of T2DM for ≥1 y, glycated hemoglobin (HbA1c) between 7.0% and 9.5%, and stable on oral antidiabetic medications for ≥3 mo before enrollment. Exclusion criteria included insulin therapy, pregnancy or lactation, chronic kidney disease (stage ≥3), active liver disease, and known allergy to cookie ingredients.

A total of 246 participants were enrolled and randomly allocated into 4 parallel groups: placebo, 5% *G. procumbens*, 10% *G. procumbens*, and 20% *G. procumbens*. The allocation sequence was generated using computer-based permuted block randomization (block size = 4) by an independent statistician who had no role in participant recruitment, intervention delivery, or outcome assessment. Eligible participants were screened and enrolled by research nurses at the participating community health centers. Allocation concealment was maintained using sequentially numbered, sealed, opaque envelopes prepared by the independent statistician. After a participant had completed enrollment and baseline assessment, the next sequential envelope was opened by the study coordinator, who assigned the participant to the indicated treatment group.

To maintain blinding, placebo and active cookies were provided in identical packaging and were standardized as closely as possible in appearance, size, texture, and flavor. Participants and laboratory personnel responsible for biomarker assessment were unaware of treatment allocation. Laboratory samples were identified using participant codes rather than treatment-group labels throughout laboratory processing and outcome assessment.

### Sample size calculation

The sample size was determined a priori based on HbA1c as the primary outcome. On the basis of previous randomized clinical trial data in adults with type 2 diabetes, an anticipated between-group difference of ∼0.44 percentage points in HbA1c, with a common SD of 0.80%, was used for the calculation [14]. Assuming a 2-sided significance level of α = 0.05 and 80% statistical power, a minimum of 53 evaluable participants per group was required. To allow for anticipated attrition, the target enrollment was increased to ∼60 participants per group, corresponding to a minimum total sample of 240 participants across the 4 study groups. The final enrollment of 246 participants therefore exceeded the minimum target sample size.

### Preparation and standardization of the functional cookies

Functional cookies (GlucoBites) were developed at the Pharmacy Laboratory, Faculty of Pharmacy, Gadjah Mada University, using a standardized production protocol to ensure consistency in composition, stability, and bioactive content across all study doses. The core ingredients included wheat flour, *G. procumbens* leaf extract, xylitol, olive oil, egg yolk, and baking powder [15], [16]. The nutritional composition of each cookie formulation was characterized in terms of energy, carbohydrate, protein, total fat, and dietary fiber. Nutritional values were expressed per 25-g cookie and for the prescribed daily intake of 2 cookies (50 g/d). The complete nutritional composition of the placebo and 5%, 10%, and 20% *G. procumbens* formulations is presented in Supplemental Table 1.

*G. procumbens* leaves were washed, oven-dried at 45°C–50°C for 48 h, and milled into fine powder. Extraction was performed using 70% ethanol maceration for 72 h, followed by filtration and concentration using a rotary evaporator under reduced pressure at 40°C–45°C to obtain a semisolid polyphenol-rich extract. The crude extract was stored in amber vials at 4°C until use [17].

To characterize the chemical composition of the intervention and distinguish constituents associated with *G. procumbens* from those originating from the background cookie matrix, comparative chemical profiling was performed on 3 materials: the pure *G. procumbens* extract, the placebo cookie, and the 5%, 10%, and 20% *G. procumbens* cookie formulation. Volatile and semivolatile constituents were analyzed by GC-MS under identical analytical conditions. Compounds were tentatively identified by comparison of their mass spectra with the instrument spectral library, and their relative abundance was expressed as percentage peak area. The placebo cookie served as a matrix control; therefore, compounds detected in both placebo and active cookie formulations were not attributed exclusively to *G. procumbens*. Comparative GC-MS profiles are presented in Figure 1A, individual chromatograms are provided in Supplemental Figure 1, and relative peak areas of selected compounds are summarized in Supplemental Table 2.

**FIGURE 1:**
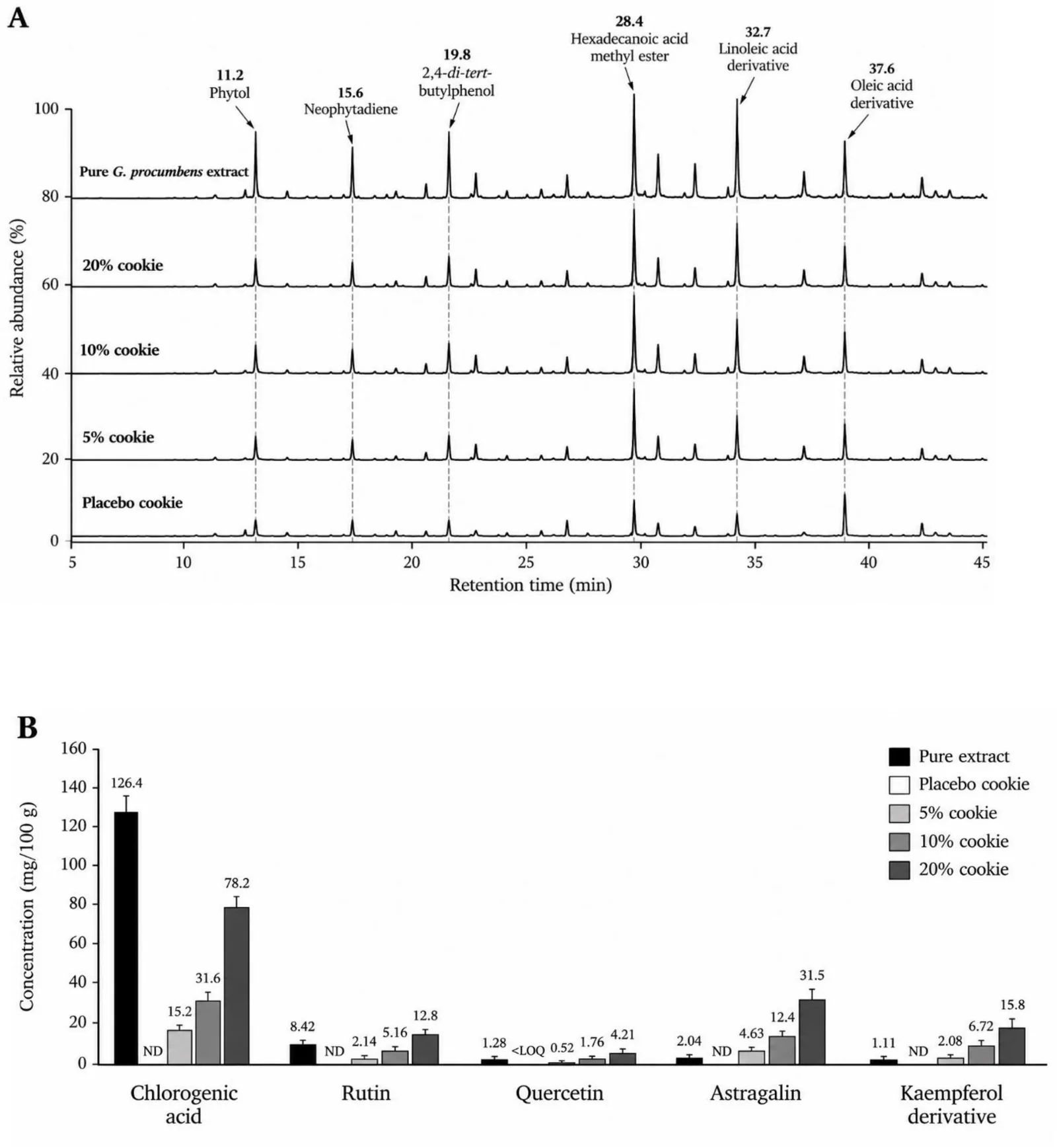
Comparative phytochemical characterization of *Gynura procumbens* extract and cookie formulations. (A) GC-MS profiles of the pure extract, placebo cookie, and 5%, 10%, and 20% *G. procumbens* cookie formulations. (B) HPLC quantification of selected phytochemical markers across the corresponding cookie formulations. Values are mean ± SD (*n* = 3). ND, not detected; <LOQ, below the limit of quantification.

Because GC-MS provides limited characterization of nonvolatile polyphenols, complementary HPLC analysis was performed to quantify selected phytochemical markers, including chlorogenic acid, rutin, quercetin, astragalin, and kaempferol-related compounds. These markers were evaluated in the pure extract, placebo cookie, and the 5%, 10%, and 20% *G. procumbens* cookie formulation to characterize phytochemical content across the full administered concentration range and to determine whether extract-associated constituents remained detectable after incorporation and baking. Chemical analyses were performed in replicate, and quantitative HPLC results are presented as mean ± SD (Figure 1B) [18].

To evaluate dose–response relationships, 4 standardized formulations were prepared: 0% (placebo), 5% *G. procumbens* extract per 100-g flour base, 10% extract, and 20% extract. The 3 active concentrations were selected to represent low, intermediate, and high levels of extract incorporation based on previous experimental evidence suggesting dose-related biologic activity of *G. procumbens* and preliminary formulation testing. During product development, formulations containing 5%, 10%, 20%, and 25% extract were evaluated for technological properties and sensory acceptability. Preliminary sensory testing was conducted among 30 untrained adult panelists using a 9-point hedonic scale (1 = dislike extremely to 9 = like extremely), with a mean overall acceptability score of ≥6 predefined as acceptable. The 5%, 10%, and 20% formulations achieved mean overall acceptability scores of 8.0 ± 0.6, 7.8 ± 0.7, and 7.2 ± 0.8, respectively, whereas the 25% formulation scored 5.6 ± 1.1. The lower acceptability of the 25% formulation was primarily related to a more pronounced bitter/herbal aftertaste, darker green appearance, and drier texture. Accordingly, the 20% formulation was selected as the highest technologically and sensorially acceptable concentration for the clinical trial. Detailed sensory evaluation results are presented in Supplemental Table 3. All formulations were matched in appearance, color, texture, and taste as closely as technically feasible to maintain participant blinding. In the placebo version, extract was replaced with an equivalent amount of wheat flour.

The mixing and homogenization process was carried out by separating dry and wet ingredients before combining them using a standardized mechanical mixer for 8–10 min to ensure complete uniformity. The dough was portioned using a calibrated mold in 25-g units to ensure identical weight and caloric content across batches. The baking procedure followed a controlled thermal protocol adapted from the functional-food stability method reported by Setyawati et al. (2025) [19], in which cookies were baked in a convection oven at 158°C for 20 min. After baking, selected phytochemical markers were assessed in the final placebo and 5%, 10%, and 20% *G. procumbens* cookie formulations to evaluate their detectability across the intervention products. After baking, cookies were cooled at room temperature (25°C–27°C) for ∼45 min before packaging.

Each cookie was vacuum-packed in food-grade polyethylene pouches to minimize oxidation and moisture exposure and stored at 4°C. All products were used within 6 wk to maintain phytochemical stability. Quality control procedures included routine testing of moisture content, weight uniformity, microbial contamination, and stability indicators (color, texture, rancidity) [20]. All 4 formulations (0%, 5%, 10%, and 20%) were produced within the same controlled batch process.

### Intervention

All participants consumed 2 cookies per day (2 × 25 g/d; total 50 g/d) for 12 wk. Aside from the varying concentrations of *G. procumbens* extract, all cookie formulations were designed to be approximately isocaloric and nutritionally comparable in terms of carbohydrate, protein, and fat content, and were standardized in size, appearance, texture, and flavor. The nutritional composition of each cookie formulation was characterized in terms of energy, carbohydrate, protein, total fat, and dietary fiber. Nutritional values were expressed per 25-g cookie and for the prescribed daily intake of 2 cookies (50 g/d). Per 25-g cookie, the placebo formulation provided ∼108 kcal, 15.4-g carbohydrate, 2.1-g protein, 4.3-g total fat, and 1.0 g dietary fiber. The 5% *G. procumbens* formulation provided ∼107 kcal, 15.2 g carbohydrate, 2.1 g protein, 4.3 g total fat, and 1.2 g dietary fiber; the 10% formulation provided ∼107 kcal, 15.0 g carbohydrate, 2.2 g protein, 4.3 g total fat, and 1.4 g dietary fiber; and the 20% formulation provided ∼106 kcal, 14.7 g carbohydrate, 2.2 g protein, 4.3 g total fat, and 1.8 g dietary fiber. Accordingly, the prescribed daily intake of 2 cookies provided ∼212–216 kcal/d, 29.4–30.8 g carbohydrate/d, 4.2–4.4 g protein/d, 8.6 g total fat/d, and 2.0–3.6 g dietary fiber/d. Thus, the 4 formulations were approximately isocaloric and generally comparable in macronutrient composition, whereas dietary fiber increased modestly with increasing *G. procumbens* concentration. On the basis of a 2000-kcal reference diet, the prescribed cookie intake contributed ∼10.6%–10.8% of daily energy requirements. The complete nutritional composition of the placebo and 5%, 10%, and 20% *G. procumbens* formulations is presented in Supplemental Table 1.

### Description of the FUNCARE model

The FUNCARE model was structured into 3 interrelated domains including biological regulation, behavioral empowerment, and holistic nursing integration and was designed to operationalize the biological effects of *G. procumbens* within a behavioral nursing framework that emphasized education, motivation, and adherence [21]. Implementation followed 3 sequential phases. In the preparation phase, nurses received standardized training on the biological mechanisms of *G. procumbens*, diabetes education principles, and motivational interviewing techniques, supported by the development of uniform educational materials, monitoring logbooks, and telecoaching protocols to ensure intervention fidelity [22]. During the implementation phase, participants attended 4 in-person coaching sessions at weeks 0, 2, 6, and 10, each lasting 60–90 min and covering diabetes pathophysiology, oxidative stress and nutrition, portion control, physical activity guidance, individualized SMART goal setting, and problem-solving discussions to address behavioral barriers [23]. Between these visits, nurses conducted biweekly telecoaching via phone or WhatsApp to monitor adherence to cookie consumption, review dietary logs, and provide ongoing motivational reinforcement. These dietary logs were used as behavioral coaching tools and were not designed as validated quantitative dietary assessment instruments. Background dietary intake was not standardized or systematically quantified using repeated 24-h dietary recalls or food-frequency assessment during the intervention. In the evaluation phase at week 12, nurses reassessed biological and behavioral outcomes, verified adherence through cookie package counts and logbook reviews, and assessed patient satisfaction and safety using structured questionnaires, forming a comprehensive and integrated model of nurse-led functional nutrition care.

### Outcome measures

Outcome measures were evaluated at baseline and at week 12. The primary outcome was the change in HbA1c (%), reflecting long-term glycemic control. Secondary outcomes included fasting plasma glucose (FPG), fasting insulin concentration, insulin resistance assessed using the HOMA-IR index, BMI, systolic blood pressure (SBP), diastolic blood pressure (DBP), total cholesterol, and vascular biomarkers consisting of the ankle-brachial index (ABI) and the matrix metalloproteinase-9 (MMP-9)/tissue inhibitor of metalloproteinase-1 (TIMP-1) ratio. Fasting insulin was measured at both time points and was used together with FPG to calculate HOMA-IR. Behavioral endpoints, including adherence to cookie consumption and patient satisfaction, were measured through structured questionnaires. Safety outcomes included the occurrence and severity of adverse events, intervention discontinuation because of adverse events, and laboratory indicators of hepatic and renal function. Adverse events were monitored throughout the 12-wk intervention using structured symptom assessments and tolerability reports. Laboratory safety parameters were assessed at baseline and week 12. Both groups received the FUNCARE nurse-led coaching program as part of standard care throughout the study; the delivery procedures for this program followed a previously standardized protocol to maintain intervention fidelity.

### Data collection procedures

Venous blood samples were obtained in the morning after an overnight fast of 8–10 h, processed within 2 h, and analyzed in a single certified clinical laboratory. FPG was measured using the glucose oxidase–peroxidase enzymatic method on an automated chemistry analyzer (Cobas c311, Roche Diagnostics) [24]. Total cholesterol was quantified in serum using an enzymatic colorimetric cholesterol oxidase–peroxidase method with the Cholesterol Gen.2 reagent kit (Roche Diagnostics GmbH) on the Cobas c311 automated chemistry analyzer, according to the manufacturer’s instructions. Total cholesterol concentrations were expressed in mg/dL. HbA1c was quantified using HPLC with the NGSP-certified Bio-Rad D-100 system (Bio-Rad Laboratories) [25]. Serum insulin, MMP-9, and TIMP-1 concentrations were determined using quantitative sandwich ELISA kits (R&D Systems), with absorbance read at 450 nm on a BioTek ELx808 microplate reader [26], [27]. Insulin resistance was calculated using the HOMA-IR formula: [fasting glucose (mg/dL) × fasting insulin (μU/mL)] / 405 [28]. Vascular function was assessed by measuring the ABI using an automated vascular Doppler (Omron HEM-907) following standardized American Heart Association procedures [29]. All laboratory assays were conducted in duplicate, and technicians were blinded to group allocation.

Safety was assessed throughout the 12-wk intervention. At each scheduled contact, participants were systematically asked about any new or worsening symptoms, including gastrointestinal complaints, and the onset, severity, duration, management, and potential relationship to the study product were documented. Serious adverse events and adverse events resulting in intervention discontinuation were also recorded.

Hepatic and renal safety were assessed at baseline and week 12. Serum aspartate aminotransferase (AST) and alanine aminotransferase (ALT) were measured using kinetic enzymatic methods based on International Federation of Clinical Chemistry recommendations with commercial AST and ALT reagent kits (Roche Diagnostics GmbH) on a Cobas c311 automated chemistry analyzer (Roche Diagnostics). Serum creatinine was measured using the Creatinine Jaffé Gen.2 reagent (Roche Diagnostics GmbH) on the same analyzer. Estimated glomerular filtration rate was calculated from serum creatinine using the 2021 Chronic Kidney Disease Epidemiology Collaboration (CKD-EPI) creatinine equation. All laboratory measurements were performed in the same certified clinical laboratory using standardized preanalytical and analytical procedures. Laboratory values were evaluated for clinically significant abnormalities according to the reference ranges used by the participating laboratory. Adherence to cookie consumption was monitored through daily logbooks, midweek verification during coaching calls, and counting of returned empty packages at each follow-up visit.

### Statistical analysis

Statistical analyses were conducted using SPSS version 26.0 (IBM Corp). Continuous variables are presented as means ± SDs, whereas categorical variables are presented as frequencies and percentages. Baseline characteristics were summarized descriptively and compared across groups using 1-way analysis of variance for continuous variables and the chi-square test or Fisher’s exact test for categorical variables, as appropriate.

The primary analysis of intervention outcomes was based on between-group comparisons using analysis of covariance (ANCOVA). For each outcome, the week-12 value was entered as the dependent variable, treatment group (placebo, 5%, 10%, and 20% *G. procumbens*) as the fixed factor, and the corresponding baseline value as a covariate. Because BMI and duration of diabetes differed significantly across groups at baseline, both variables were included as additional covariates in the adjusted models. Adjusted marginal means at week 12 were estimated for each treatment group. Comparisons of each active-treatment group with placebo were performed using Dunnett-adjusted contrasts to account for multiple comparisons against a common control. Results are reported as adjusted marginal means, adjusted mean differences (AMDs), corresponding 95% confidence intervals, and Dunnett-adjusted *P* values. Partial eta-squared (partial η^2^) was used to estimate the magnitude of the overall treatment effect. A linear contrast across increasing *G. procumbens* concentrations was additionally examined to assess evidence of a dose–response relationship.

Within-group changes from baseline to week 12 were examined as supplementary descriptive analyses using paired *t* tests for normally distributed variables and Wilcoxon signed-rank tests when normality assumptions were not met. These within-group analyses were not used as the primary basis for treatment-effect inference and are reported separately in Supplemental Table 4. Percentage change was calculated as [(postintervention value − baseline value) / baseline value] × 100 for descriptive purposes. For the ANCOVA models, assumptions were evaluated by examining residual normality, homogeneity of variance, and homogeneity of regression slopes. Because BMI and duration of diabetes differed significantly across groups at baseline, both variables were included as additional covariates in the adjusted ANCOVA models. Dietary intake was not included as a covariate because repeated quantitative dietary intake data were not systematically collected during follow-up. Therefore, potential changes in habitual energy and macronutrient intake could not be statistically adjusted for in the outcome models. A 2-tailed *P* value <0.05 was considered statistically significant.

The primary outcome analyses were conducted using a complete-case approach. Participants with available baseline and week-12 outcome data were analyzed according to their originally assigned treatment groups. Three participants who withdrew consent during follow-up did not have complete week-12 outcome data and were therefore excluded from the complete-case analyses. No imputation of missing outcome data was performed. Consequently, the final analyses included 243 of the 246 randomly divided participants.

### Ethical considerations

This study was reviewed and approved by the Health Research Ethics Committee of the Faculty of Nursing, Hasanuddin University, Makassar, Indonesia (Approval No. 527/UN4.18.3/TP.01.02/2025; 14 July 2025). The review was conducted through a full board process and declared ethically feasible for implementation until 14 July 2026. Written informed consent was obtained from all participants before enrollment and before any study-specific procedures were performed. Participation was voluntary, and participants were informed of their right to withdraw from the study at any time without affecting their usual clinical care.

The trial was registered at clinicaltrials.gov (identifier NCT07151833). The registration was first submitted on 22 August 2025 and publicly posted on 3 September 2025. The first participant was enrolled on 20 September 2025; therefore, trial registration preceded enrollment of the first participant. All study procedures were conducted in accordance with the principles of the Declaration of Helsinki and applicable institutional and local ethical guidelines for biomedical research involving human participants.

## Results

### Participant flow

A total of 288 individuals were assessed for eligibility across the participating community health centers. Of these, 44 individuals were excluded, with 31 not meeting the inclusion criteria and 11 declining to participate. A total of 246 eligible participants were subsequently randomly divided into 4 groups: placebo (*n* = 61), 5% *G. procumbens* (*n* = 61), 10% *G. procumbens* (*n* = 62), and 20% *G. procumbens* (*n* = 62). Thus, 185 participants were allocated to the 3 active intervention groups and 61 to the placebo group.

During follow-up, 2 participants from the therapeutic cookie groups and 1 participant from the placebo group withdrew consent and were lost to follow-up. Consequently, 183 participants in the active intervention groups and 60 participants in the placebo group completed follow-up and were included in the final analysis, yielding a total analyzed sample of 243 participants. No additional participants were excluded from the analysis. The full CONSORT flow diagram is presented in Figure 2.

**FIGURE 2:**
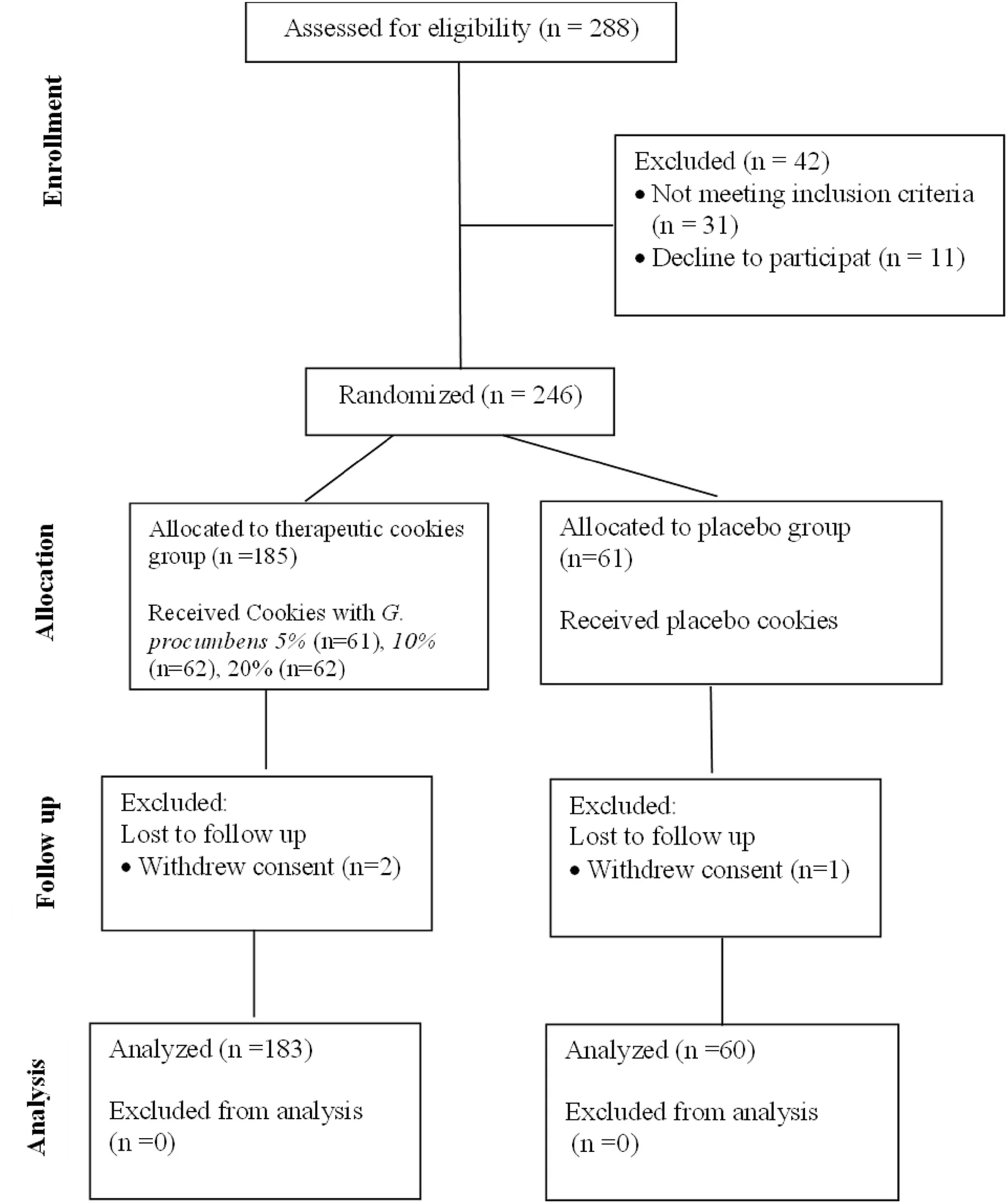
CONSORT flow diagram showing participant enrollment, allocation, follow-up, and analysis across placebo and *G. procumbens* cookie groups.

### Participant characteristics

Baseline characteristics of participants across the 4 study groups are presented in Table 1. Overall, demographic and clinical profiles were comparable between groups. The mean age ranged from 53.3 ± 3.7 to 61.3 ± 4.8 y, with no statistically significant difference across groups (*P* = 0.55). The proportion of female participants was similar in all groups (56%–60%, *P* = 0.88). SBP and DBP values were also comparable, with no significant between-group variation (SBP, *P* = 0.72; DBP, *P* = 0.87).

**TABLE 1:** Baseline characteristics of participants

Characteristic	Placebo (*n* = 60)	5% *G. procumbens* (*n* = 61)	10% *G. procumbens* (*n* = 61)	20% *G. procumbens* (*n* = 61)	*P* value
Age (y)	53.3 ± 3.7	56.9 ± 4.9	54.8 ± 5.1	61.3 ± 4.8	0.55
Female (%)	57	59	56	60	0.88
Duration of diabetes (y)	13.6 ± 5.1	9.2 ± 3.7	6.2 ± 2.0	11.2 ± 4.6	0.03^1^
BMI (kg/m^2^)	28.1 ± 2.8	25.3 ± 3.6	25.7 ± 3.1	26.4 ± 4.1	0.04^1^
Systolic BP (mmHg)	130.8 ± 10.4	131.2 ± 9.8	129.9 ± 10.2	131.4 ± 9.5	0.72
Diastolic BP (mmHg)	82.1 ± 7.8	81.7 ± 4.5	82.4 ± 6.7	81.9 ± 5.9	0.87
Fasting plasma glucose (mg/dL)	144.7 ± 21.6	155.1 ± 22.3	154.0 ± 21.9	147.2 ± 22.4	0.81
HbA1c (%)	7.4 ± 0.4	7.6 ± 1.6	7.4 ± 0.3	7.8 ± 1.5	0.89
HOMA-IR	4.8 ± 0.9	4.9 ± 1.0	4.7 ± 0.8	4.9 ± 0.9	0.77
Ankle-brachial index	0.89 ± 0.12	0.93 ± 0.09	0.91 ± 0.11	0.87 ± 0.05	0.80
Total cholesterol (mg/dL)	221.6 ± 31.4	214.9 ± 29.7	199.2 ± 28.1	218.3 ± 27.5	0.44

Data are presented as mean ± SD unless otherwise indicated. *P* values were obtained using 1-way analysis of variance.

Abbreviations: BP, blood pressure; HbA1c, glycated hemoglobin.

1 *P* < 0.05 indicates statistically significant difference among groups. Because BMI and duration of diabetes differed significantly across groups at baseline, both variables were included as additional covariates in the adjusted ANCOVA models.

Duration of diabetes and BMI differed significantly across groups (*P* = 0.03 and *P* = 0.04, respectively). These variables were therefore included as additional covariates in the adjusted outcome analyses. Baseline metabolic parameters, including FPG, HbA1c, HOMA-IR, ABI, and total cholesterol, did not differ significantly across groups (all *P* > 0.05). Duration of diabetes and BMI differed significantly across groups (*P* = 0.03 and *P* = 0.04, respectively). These variables were therefore included as additional covariates in the adjusted outcome analyses.

All baseline metabolic parameters, including FPG (144.7–155.1 mg/dL, *P* = 0.81), HbA1c (7.4%–7.8%, *P* = 0.89), HOMA-IR (4.7–4.9, *P* = 0.77), ABI (0.87–0.93, *P* = 0.80), and total cholesterol (199.2–221.6 mg/dL, *P* = 0.44), were statistically similar between groups.

### Glycemic outcomes

Figure 3A illustrates the participant-level distribution of FPG levels across the 4 study groups at baseline and week 12. As shown in Table 2, after adjustment for baseline FPG, BMI, and duration of diabetes, no significant overall treatment-group effect was observed for week-12 FPG (ANCOVA, *P* = 0.61; partial η^2^ = 0.008). The adjusted mean FPG values were 160.2 mg/dL in the placebo group, 159.1 mg/dL in the 5% group, 158.0 mg/dL in the 10% group, and 156.8 mg/dL in the 20% group. Compared with placebo, none of the active formulations showed a significant difference after Dunnett adjustment (5%: AMD, −1.1 mg/dL; 95% CI: −8.0, 5.8; *P* = 0.94; 10%: AMD, −2.2 mg/dL; 95% CI: −9.1, 4.7; *P* = 0.82; 20%: AMD, −3.4 mg/dL; 95% CI: −10.3, 3.5; *P* = 0.49). There was also no evidence of a linear dose–response trend for FPG (*P* = 0.28).

**FIGURE 3:**
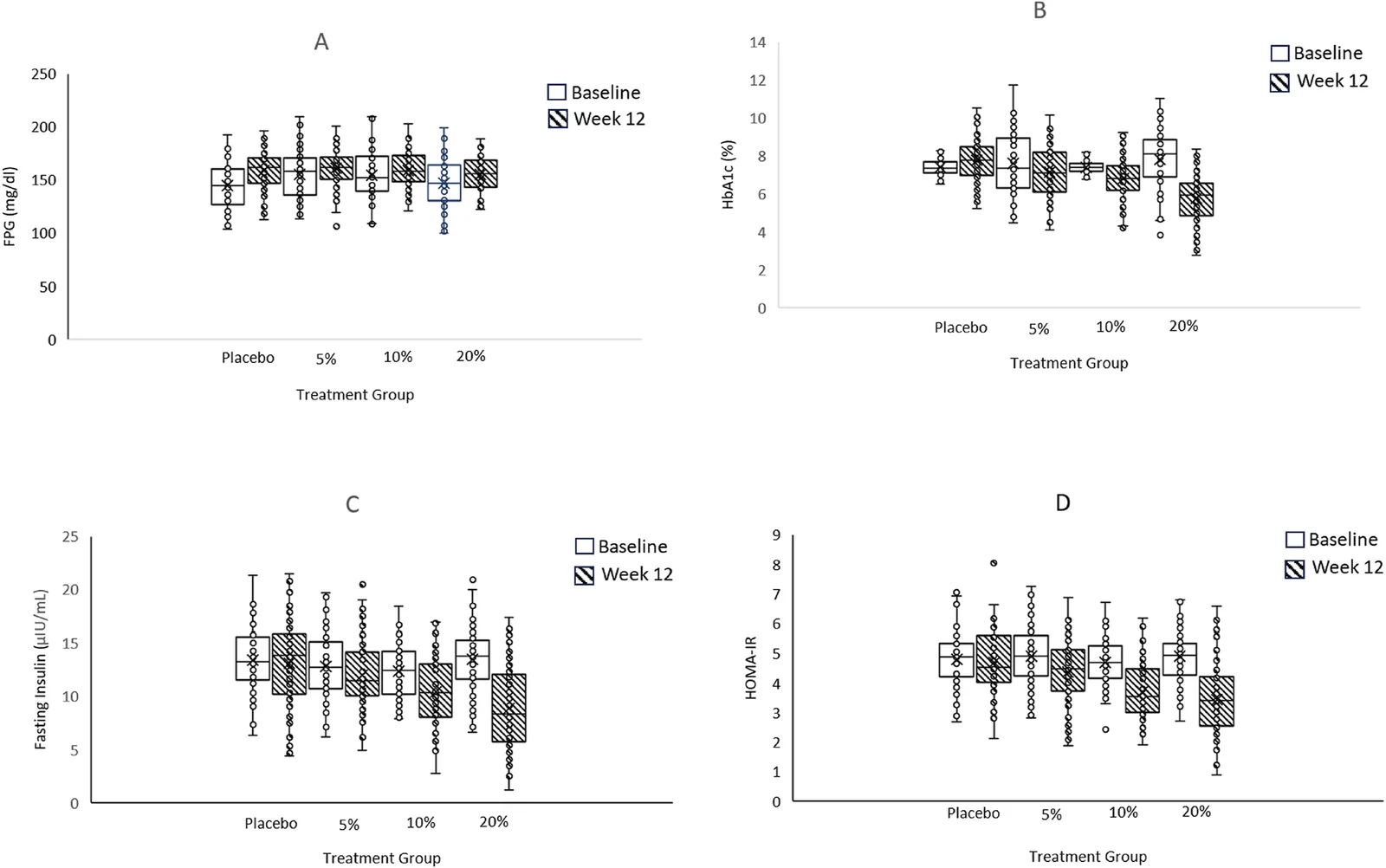
Distribution of glycemic and insulin-related outcomes across treatment groups at baseline and week 12. Box-and-whisker plots with individual participant observations show (A) fasting plasma glucose, (B) HbA1c, (C) fasting insulin, and (D) HOMA-IR in the placebo, 5%, 10%, and 20% *G. procumbens* groups. Within each treatment group, the open box represents baseline, and the hatched box represents week 12. Boxes indicate the interquartile range, horizontal lines indicate medians, and individual points represent participant-level observations. HbA1c, glycated hemoglobin.

**TABLE 2:** Adjusted between-group effects on vascular and biomarker outcomes at week 12

Outcome	Group	Baselinemean ± SD	Week-12mean ± SD	Adjustedweek-12 mean	Adjusted differencevs. placebo (95% CI)	Adjusted*P* value
ABI	Placebo	0.88 ± 0.06	0.89 ± 0.05	0.890	Reference	—
	5% *G. procumbens*	0.90 ± 0.12	0.92 ± 0.16	0.916	+0.026 (−0.008, 0.060)	0.18
	10% *G. procumbens*	0.89 ± 0.08	0.93 ± 0.17	0.931	+0.041 (0.007, 0.075)	0.012
	20% *G. procumbens*	0.87 ± 0.13	0.94 ± 0.09	0.942	+0.052 (0.018, 0.086)	0.001
MMP-9 (ng/mL)	Placebo	322.4 ± 61.2	318.5 ± 57.2	318.5	Reference	—
	5% *G. procumbens*	335.8 ± 68.9	320.2 ± 62.7	320.2	+1.7 (−22.6, 26.0)	0.99
	10% *G. procumbens*	341.2 ± 72.3	309.5 ± 59.1	309.5	−9.0 (−33.3, 15.3)	0.68
	20% *G. procumbens*	338.3 ± 66.4	286.1 ± 55.8	286.1	−32.4 (−56.7, −8.1)	0.004
TIMP-1 (ng/mL)	Placebo	169.4 ± 29.9	168.8 ± 27.3	168.8	Reference	—
	5% *G. procumbens*	172.3 ± 31.1	175.8 ± 29.2	175.8	+7.0 (−4.9, 18.9)	0.36
	10% *G. procumbens*	170.8 ± 33.2	178.2 ± 33.3	178.2	+9.4 (−2.5, 21.3)	0.15
	20% *G. procumbens*	172.4 ± 28.4	187.7 ± 31.4	187.7	+18.9 (7.0, 30.8)	0.001
MMP-9/TIMP-1 ratio	Placebo	1.90 ± 0.42	1.89 ± 0.40	1.89	Reference	—
	5% *G. procumbens*	1.95 ± 0.47	1.83 ± 0.33	1.83	−0.06 (−0.20, 0.08)	0.56
	10% *G. procumbens*	2.01 ± 0.49	1.74 ± 0.38	1.74	−0.15 (−0.29, −0.01)	0.032
	20% *G. procumbens*	1.98 ± 0.45	1.54 ± 0.36	1.55	−0.34 (−0.48, −0.20)	<0.001

Values are presented as mean ± SD unless otherwise indicated. Adjusted week-12 means were estimated using analysis of variance with the corresponding baseline outcome, BMI, and duration of diabetes as covariates. Adjusted mean differences represent Dunnett-adjusted comparisons of each active-treatment group with placebo.

Abbreviations: ABI, ankle-brachial index; CI, confidence interval; MMP-9, matrix metalloproteinase-9; TIMP-1, tissue inhibitor of metalloproteinase-1.

Figure 3B shows the participant-level distribution of HbA1c levels at baseline and week 12 across the study groups. In contrast to FPG, the adjusted between-group analysis demonstrated a significant overall treatment-group effect on week-12 HbA1c (ANCOVA, *P* < 0.001; partial η^2^ = 0.34) (Table 2). The adjusted mean HbA1c values were 7.76% in the placebo group, 7.17% in the 5% group, 6.92% in the 10% group, and 5.63% in the 20% group. Compared with placebo, HbA1c was lower by 0.59 percentage points in the 5% group (95% CI: −1.01, −0.17; Dunnett-adjusted *P* = 0.006), 0.84 percentage points in the 10% group (95% CI: −1.26, −0.42; *P* < 0.001), and 2.13 percentage points in the 20% group (95% CI: −2.56, −1.70; *P* < 0.001). A significant linear dose–response trend was observed across increasing *G. procumbens* concentrations (*P* < 0.001). The participant-level distributions shown in Figure 3B visually demonstrate a similar dose-related pattern at week 12.

Figure 3C shows the participant-level distribution of fasting insulin concentrations across the study groups at baseline and week 12. After adjustment for baseline fasting insulin, BMI, and duration of diabetes, a significant overall treatment-group effect was observed at week 12 (ANCOVA, *P* < 0.001; partial η^2^ = 0.16). Compared with placebo, adjusted fasting insulin concentrations were significantly lower in the 10% group (AMD, −2.4 μIU/mL; 95% CI: −3.6, −1.2; Dunnett-adjusted *P* < 0.001) and in the 20% group (AMD, −3.9 μIU/mL; 95% CI: −5.1, −2.7; *P* < 0.001), whereas the difference in the 5% group was not statistically significant (AMD, −0.9 μIU/mL; 95% CI: −2.1, 0.3; *P* = 0.18). The distribution shown in Figure 3C is consistent with a greater reduction in fasting insulin in the higher-dose groups, particularly the 20% *G. procumbens* group.

Figure 3D shows the participant-level distribution of HOMA-IR values across the study groups at baseline and week 12. The adjusted analysis showed a significant overall treatment-group effect on HOMA-IR (ANCOVA, *P* < 0.001; partial η^2^ = 0.19) (Table 2). Adjusted mean HOMA-IR values were 4.62 in the placebo group, 4.38 in the 5% group, 3.82 in the 10% group, and 3.39 in the 20% group. Compared with placebo, the difference was not statistically significant in the 5% group (AMD, −0.24; 95% CI: −0.63, 0.15; *P* = 0.31), whereas significantly lower HOMA-IR values were observed in the 10% group (AMD, −0.80; 95% CI: −1.19, −0.41; *P* < 0.001) and the 20% group (AMD, −1.23; 95% CI: −1.62, −0.84; *P* < 0.001). A significant linear dose–response trend was also observed for HOMA-IR (*P* < 0.001), consistent with the progressively lower values observed in the higher-dose groups in Figure 3C.

Fasting insulin concentrations showed a significant between-group treatment effect at week 12 (ANCOVA, *P* < 0.001, partial η^2^ = 0.16; Table 3). Compared with placebo, adjusted fasting insulin was 2.4 μIU/mL lower in the 10% *G. procumbens* group (95% CI: −3.6, −1.2; Dunnett-adjusted *P* < 0.001) and 3.9 μIU/mL lower in the 20% group (95% CI: −5.1, −2.7; *P* < 0.001). The 5% group did not significantly differ from placebo. This pattern indicates that the improvement in HOMA-IR in the higher-dose groups was accompanied primarily by lower fasting insulin rather than substantial changes in fasting glucose.

**TABLE 3:** Cardiometabolic measures at baseline and week 12 across treatment groups

Outcome	Group	Baseline,mean ± SD	Week 12,mean ± SD	Adjustedweek-12mean (95% CI)	Adjusteddifferencevs. placebo (95% CI)	Dunnett-adjusted *P*	OverallANCOVA *P*;partial η^2^
BMI (kg/m^2^)	Placebo	28.1 ± 2.8	27.9 ± 2.7	26.6 (26.2, 27.0)	Reference	—	0.42; 0.012
	5% *G. procumbens*	25.3 ± 3.6	25.1 ± 3.4	26.5 (26.1, 26.9)	−0.1 (−0.7, 0.5)	0.97	
	10% *G. procumbens*	25.7 ± 3.1	25.4 ± 3.0	26.3 (25.9, 26.7)	−0.3 (−0.9, 0.3)	0.55	
	20% *G. procumbens*	26.4 ± 4.1	26.0 ± 3.8	26.1 (25.7, 26.5)	−0.5 (−1.1, 0.1)	0.14	
SBP (mmHg)	Placebo	130.8 ± 10.4	130.2 ± 10.1	130.1 (127.8, 132.4)	Reference	—	0.08; 0.028
	5% *G. procumbens*	131.2 ± 9.8	129.6 ± 9.3	129.2 (126.9, 131.5)	−0.9 (−4.1, 2.3)	0.83	
	10% *G. procumbens*	129.9 ± 10.2	128.3 ± 9.5	128.4 (126.1, 130.7)	−1.7 (−4.9, 1.5)	0.46	
	20% *G. procumbens*	131.4 ± 9.5	127.4 ± 9.1	127.1 (124.8, 129.4)	−3.0 (−6.2, 0.2)	0.08	
DBP (mmHg)	Placebo	82.1 ± 7.8	81.8 ± 7.3	81.7 (80.1, 83.3)	Reference	—	0.31; 0.015
	5% *G. procumbens*	81.7 ± 4.5	81.0 ± 4.7	81.1 (79.5, 82.7)	−0.6 (−2.8, 1.6)	0.87	
	10% *G. procumbens*	82.4 ± 6.7	80.6 ± 6.2	80.5 (78.9, 82.1)	−1.2 (−3.4, 1.0)	0.45	
	20% *G. procumbens*	81.9 ± 5.9	79.9 ± 5.6	80.0 (78.4, 81.6)	−1.7 (−3.9, 0.5)	0.18	
Fasting insulin(μIU/mL)	Placebo	13.4 ± 3.1	13.0 ± 3.0	12.9 (12.1, 13.7)	Reference	—	<0.001; 0.16
	5% *G. procumbens*	12.8 ± 3.0	11.9 ± 2.8	12.0 (11.2, 12.8)	−0.9 (−2.1, 0.3)	0.18	
	10% *G. procumbens*	12.4 ± 2.8	10.3 ± 2.5	10.5 (9.7, 11.3)	−2.4 (−3.6, −1.2)	<0.001	
	20% *G. procumbens*	13.5 ± 3.2	8.8 ± 2.2	9.0 (8.2, 9.8)	−3.9 (−5.1, −2.7)	<0.001	
Total cholesterol(mg/dL)	Placebo	221.6 ± 31.4	219.8 ± 30.6	216.7 (209.8, 223.6)	Reference	—	0.09; 0.027
	5% *G. procumbens*	214.9 ± 29.7	211.5 ± 28.4	213.1 (206.2, 220.0)	−3.6 (−13.3, 6.1)	0.70	
	10% *G. procumbens*	199.2 ± 28.1	195.6 ± 27.2	209.3 (202.4, 216.2)	−7.4 (−17.1, 2.3)	0.18	
	20% *G. procumbens*	218.3 ± 27.5	203.8 ± 26.1	207.1 (200.2, 214.0)	−9.6 (−19.3, 0.1)	0.06	

Values are presented as unadjusted mean ± SD at baseline and week 12 and adjusted week-12 means with 95% confidence intervals. Between-group comparisons were performed using ANCOVA with the corresponding baseline value, baseline BMI, and duration of diabetes as covariates. The placebo group was the reference category. Pairwise comparisons of each active-treatment group vs. placebo were adjusted using Dunnett’s procedure. Partial η^2^ represents the effect size for the overall treatment-group effect.

Abbreviations: ANCOVA, analysis of variance; DBP, diastolic blood pressure; SBP, systolic blood pressure.

### Additional cardiometabolic outcomes

Additional cardiometabolic outcomes are presented in Table 3. No significant between-group treatment effects were observed for BMI, SBP, or DBP after adjustment for baseline values, BMI, and duration of diabetes (all *P* > 0.05). Total cholesterol showed a greater numerical reduction in the higher-dose groups, particularly the 20% group, although the overall between-group effect did not reach statistical significance (ANCOVA *P* = 0.09, partial η^2^ = 0.027). These findings indicate that the clearest additional cardiometabolic response was observed for fasting insulin rather than anthropometric, blood pressure, or total cholesterol outcomes.

### Vascular and biomarker outcomes

Adjusted between-group analyses of vascular function and biomarker outcomes are presented in Table 2. After adjustment for the corresponding baseline values, BMI, and duration of diabetes, a significant overall treatment-group effect was observed for the ABI at week 12 (ANCOVA, *P* = 0.004; partial η^2^ = 0.057). The adjusted mean ABI values were 0.890, 0.916, 0.931, and 0.942 in the placebo, 5%, 10%, and 20% *G. procumbens* groups, respectively. Compared with placebo, the adjusted difference was not statistically significant for the 5% formulation (+0.026; 95% CI: −0.008, 0.060; Dunnett-adjusted *P* = 0.18), whereas significantly higher ABI values were observed in the 10% group (+0.041; 95% CI: 0.007, 0.075; *P* = 0.012) and the 20% group (+0.052; 95% CI: 0.018, 0.086; *P* = 0.001). A significant linear dose–response trend was observed for ABI (*P* = 0.001).

MMP-9 concentrations also differed across treatment groups after covariate adjustment (overall ANCOVA, *P* = 0.008; partial η^2^ = 0.049). Adjusted week-12 MMP-9 concentrations were 318.5, 320.2, 309.5, and 286.1 ng/mL in the placebo, 5%, 10%, and 20% groups, respectively. Neither the 5% group (AMD, +1.7 ng/mL; 95% CI: −22.6, 26.0; *P* = 0.99) nor the 10% group (AMD, −9.0 ng/mL; 95% CI: −33.3, 15.3; *P* = 0.68) differed significantly from placebo after Dunnett adjustment. In contrast, MMP-9 was significantly lower in the 20% group (AMD, −32.4 ng/mL; 95% CI: −56.7, −8.1; *P* = 0.004).

For TIMP-1, the adjusted analysis demonstrated an overall treatment-group effect at week 12 (ANCOVA, *P* = 0.012; partial η^2^ = 0.044). Adjusted mean TIMP-1 concentrations were 168.8, 175.8, 178.2, and 187.7 ng/mL in the placebo, 5%, 10%, and 20% groups, respectively. Compared with placebo, differences in the 5% (+7.0 ng/mL; 95% CI: −4.9, 18.9; *P* = 0.36) and 10% groups (+9.4 ng/mL; 95% CI: −2.5, 21.3; *P* = 0.15) were not statistically significant, whereas TIMP-1 was significantly higher in the 20% group (+18.9 ng/mL; 95% CI: 7.0, 30.8; *P* = 0.001).

The MMP-9/TIMP-1 ratio demonstrated a significant overall treatment-group effect (ANCOVA, *P* < 0.001; partial η^2^ = 0.14) (Table 2). Adjusted mean ratios were 1.89, 1.83, 1.74, and 1.55 in the placebo, 5%, 10%, and 20% groups, respectively. Compared with placebo, the adjusted difference was not significant in the 5% group (AMD, −0.06; 95% CI: −0.20, 0.08; *P* = 0.56), but was significant in the 10% group (AMD, −0.15; 95% CI: −0.29, −0.01; *P* = 0.032) and the 20% group (AMD, −0.34; 95% CI: −0.48, −0.20; *P* < 0.001). A significant linear dose –response trend was observed for the MMP-9/TIMP-1 ratio (*P* < 0.001).

### Adherence, acceptability, and safety

As shown in Figure 4A–C, adherence, acceptability, and safety outcomes collectively demonstrated that the cookie-based intervention was well tolerated and feasible across all study groups. Adherence remained consistently high, exceeding 90% in all groups and reaching its peak in the 20% *G. procumbens* group, indicating that increasing the proportion of the functional ingredient did not diminish daily compliance. Overall acceptability was likewise favorable, with the majority of participants rating the cookies as “very good” or “good,” reflecting positive perceptions of taste, texture, and convenience. Safety outcomes were assessed separately across the 4 treatment groups. Mild, transient gastrointestinal symptoms were reported by 2 participants (3.3%) in the placebo group, 4 (6.6%) in the 5% *G. procumbens* group, 6 (9.7%) in the 10% group, and 8 (12.9%) in the 20% group. Overall, 20 of 246 participants (8.1%) experienced mild gastrointestinal symptoms during the intervention. These symptoms occurred predominantly during the first week and resolved without specific treatment. No serious adverse events, adverse events requiring medical treatment, or intervention discontinuations attributable to the study product were observed in any group. Detailed laboratory safety parameters and adverse events by treatment group are presented in Supplemental Table 5.

**FIGURE 4:**
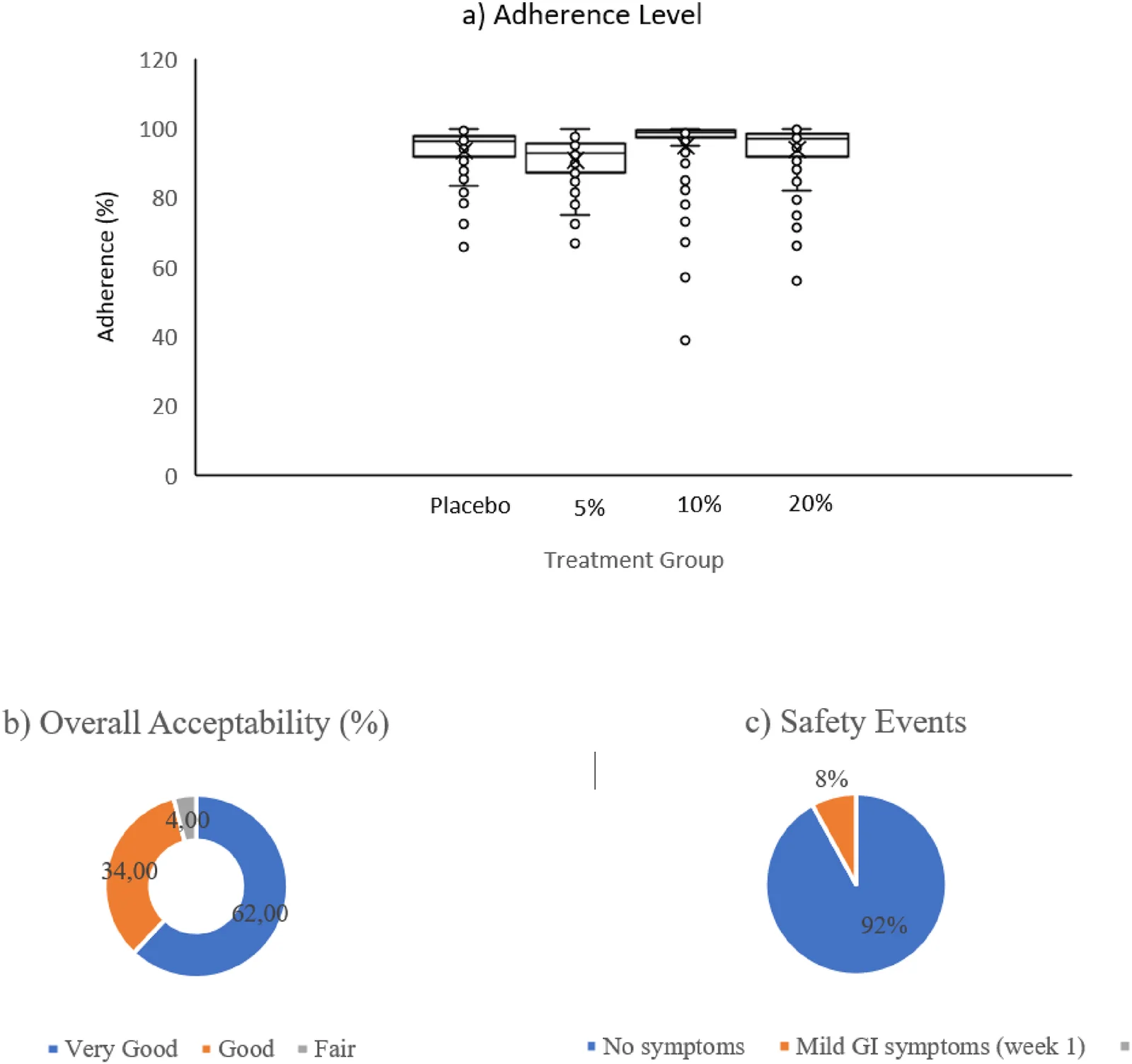
Adherence, acceptability, and safety profile of the *G. procumbens* functional nutrition intervention.

#### Feasibility indicators

Recruitment and retention outcomes also demonstrated strong feasibility of the intervention. Of the 246 randomly divided participants, 243 completed the 12-wk follow-up, corresponding to a retention rate of 98.8%. In addition, 88% of respondents indicated willingness to participate in future trials, reflecting sustained engagement and favorable perceptions of the intervention.

#### Comparative chemical characterization of the intervention materials

Comparative chemical profiling demonstrated differences in the distribution and relative abundance of selected constituents across the pure *G. procumbens* extract, placebo cookie, and 5%, 10%, and 20% active formulations (Figure 1A; Supplementary Figure 1; Supplementary Table 2). Phytol and neophytadiene were detected in the pure extract and active cookie formulations but were not detected in the placebo formulation. Their relative peak areas showed progressively greater abundance across the 5%, 10%, and 20% formulations, increasing from 0.8% to 1.6% and 2.5% for phytol, and from 0.5% to 1.0% and 1.7% for neophytadiene, respectively, supporting their association with *G. procumbens* incorporation. In contrast, hexadecanoic acid methyl ester, linoleic acid derivatives, and oleic acid derivatives were present in both the placebo and active formulations and showed comparatively smaller differences across concentrations, indicating that these compounds were predominantly associated with the background cookie matrix.

HPLC analysis provided complementary quantitative characterization of selected nonvolatile phytochemical markers across the formulations (Figure 1B). Chlorogenic acid, rutin, astragalin, and kaempferol-related compounds were not detected in the placebo formulation but were measurable in the 5%, 10%, and 20% *G. procumbens* cookies. Chlorogenic acid concentrations were 15.2, 31.6, and 78.2 mg/100 g; rutin concentrations were 2.14, 5.16, and 12.8 mg/100 g; astragalin concentrations were 4.63, 12.4, and 31.5 mg/100 g; and kaempferol-related compounds were 2.08, 6.72, and 15.8 mg/100 g in the 5%, 10%, and 20% formulations, respectively. Quercetin was below the limit of quantification in the placebo formulation and was measurable at 0.52, 1.76, and 4.21 mg/100 g in the 5%, 10%, and 20% formulations, respectively.

## Discussion

This randomized controlled trial demonstrated significant between-group effects of *G. procumbens*–enriched cookies on selected metabolic and vascular outcomes when delivered within a standardized nurse-led FUNCARE framework in adults with T2DM. After adjustment for baseline outcome values, BMI, and duration of diabetes, no significant treatment-group effect was observed for FPG. In contrast, HbA1c differed significantly across groups, with adjusted reductions relative to placebo of 0.59, 0.84, and 2.13 percentage points in the 5%, 10%, and 20% *G. procumbens* groups, respectively (Table 2; Figure 3). HOMA-IR was also significantly lower than placebo in the 10% and 20% groups, indicating greater improvement in insulin resistance at the higher concentrations. These metabolic findings are consistent with the potential for structured lifestyle and behavioral support to contribute to metabolic risk management [30]. These metabolic effects were accompanied by significant between-group differences in vascular outcomes. ABI, an established measure used in the assessment of lower-extremity peripheral arterial disease [29], was significantly higher than placebo in the 10% and 20% groups, whereas the MMP-9/TIMP-1 ratio was significantly lower in the 10% and 20% groups. Significant linear contrasts for HbA1c, HOMA-IR, ABI, and the MMP-9/TIMP-1 ratio further supported a dose-related pattern of response. Collectively, these findings suggest that increasing concentrations of *G. procumbens*, particularly the 10% and 20% formulations, may provide incremental metabolic and vascular benefits compared with placebo when delivered alongside standardized nurse-led behavioral support [30]. However, the glycemic response was not consistent across all measures, as no significant treatment-group effect was observed for FPG. Therefore, the glycemic findings should be interpreted as selective rather than uniform.

Importantly, background dietary intake was not systematically quantified throughout the 12-wk intervention. Although all groups received the same standardized nurse-led coaching, and the cookie formulations were approximately isocaloric, participants may have made concurrent changes in habitual food intake in response to dietary counseling or other factors. Such changes could have influenced glycemic and metabolic outcomes and were not available for adjustment in the present analyses. Therefore, the observed between-group differences should be interpreted in the context of potential residual dietary confounding.

Comparative chemical profiling provided a more specific characterization of the intervention product by distinguishing constituents associated with *G. procumbens* from those contributed by the background cookie matrix. GC-MS analysis showed that several lipid-derived compounds, including hexadecanoic acid methyl ester, linoleic acid derivatives, and oleic acid derivatives, were present in the placebo and all active *G. procumbens* cookie formulations, indicating that these constituents were predominantly matrix-associated and could not be attributed specifically to *G. procumbens*. In contrast, phytol and neophytadiene were detected in the pure extract and remained detectable across the 5%, 10%, and 20% cookie formulations, but were not detected in the placebo formulation. Complementary quantitative HPLC analysis further confirmed selected nonvolatile polyphenolic markers across the pure extract and the 5%, 10%, and 20% *G. procumbens* cookie formulations, whereas these markers were absent or below quantifiable levels in the placebo formulation. A concentration-related increase in selected phytochemical markers was observed across the 5%, 10%, and 20% formulations, indicating progressively greater phytochemical exposure with increasing levels of extract incorporation.

These chemical findings strengthen the biologic plausibility of the intervention and provide a more complete context for interpreting the dose-related patterns observed in selected metabolic and vascular outcomes. However, they do not establish that any individual phytochemical, or the observed phytochemical gradient itself, was responsible for the glycemic or vascular effects [31], [32], [33], [34], [35]. Previous studies suggest that lipid-derived compounds and dietary polyphenols may exert anti-inflammatory and vascular-protective effects, including modulation of matrix metalloproteinase activity and endothelial function [31], [32], [33], [34], [35], [36]. These mechanisms may be biologically consistent with the observed changes in MMP-9, TIMP-1, and ABI; however, the specific molecular pathways responsible for these clinical findings were not directly assessed in this study.

Beyond lipid-derived compounds, polyphenolic constituents associated with *G. procumbens* may also contribute to its potential metabolic effects. Experimental studies of quercetin and related polyphenols have demonstrated enhanced glucose uptake and GLUT4 translocation through pathways involved in glucose metabolism [36], [37]. The reductions in HOMA-IR observed in the 10% and 20% groups are consistent with improved insulin sensitivity; however, the specific molecular pathways underlying these changes were not directly assessed in this study. Overall, these findings provide biologic plausibility for the metabolic effects observed with the higher-dose formulations without establishing a specific molecular mechanism. [38]. Although the biomarker responses varied across outcomes, the overall pattern was consistent with the potential metabolic effects reported for *G. procumbens*. However, these clinical findings should be interpreted as supportive rather than confirmatory evidence of the underlying biologic mechanisms.

From a nursing standpoint, the FUNCARE model illustrates how biologically grounded nutritional interventions can be effectively embedded within nurse-led coaching to enhance self-management behaviors [22]. High adherence rates, exceeding 90% across all groups, and strong acceptability ratings indicate that the intervention was both feasible and well integrated into participants’ daily routines. The absence of serious adverse events and the low incidence of transient gastrointestinal discomfort further demonstrate the safety of the formulation, even at higher concentrations. Through structured coaching, nurses facilitated dietary adaptation, reinforced behavioral goals, and provided ongoing motivation, thereby providing a consistent behavioral-support context across all treatment groups. Because the same coaching was delivered to all groups, its independent contribution to the observed metabolic improvements cannot be determined from this study design. This integrative approach aligns with the nursing metaparadigm by addressing physiological, psychosocial, and behavioral dimensions of chronic disease management [39].

Compared with earlier studies, which were predominantly preclinical or observational, this trial provides a more rigorous evaluation of *G. procumbens* through standardized dosing, controlled formulation, and randomly assigned allocation. The reductions in HbA1c observed in the active-treatment groups, particularly at the 10% and 20% concentrations, were notable; however, their interpretation should be considered alongside the absence of a significant treatment effect on FPG and the potential for residual dietary confounding. Importantly, the integration of nurse-led coaching offers an added behavioral mechanism that is often missing in functional-food trials, reinforcing the translational value of combining nutrition science with nursing practice [40].

The feasibility of the FUNCARE model was further supported by operational indicators. Recruitment was completed earlier than projected, retention remained high at 91.7%, and most participants expressed willingness to join future trials. These factors demonstrate not only the acceptability of the intervention but also its potential scalability within community health systems. Nonetheless, several limitations should be acknowledged. The 12-wk duration may not fully capture long-term metabolic trajectories, and the study was not powered to detect changes in hard cardiovascular outcomes. Future research should incorporate longer follow-up, multicenter recruitment, and more granular biomarker analyses, including oxidative and inflammatory pathways. The integration of digital health tools may also enhance remote monitoring and sustain engagement.

Overall, the FUNCARE model represents a promising convergence of functional nutrition and nurse-led behavioral care. By addressing both biological and behavioral determinants of glycemic control, this model highlights the essential role of nurses in translating emerging nutritional evidence into practical, culturally relevant interventions that support metabolic health and quality of life among individuals with T2DM.

This study has several limitations. First, the 12-wk intervention period may not have been sufficient to capture longer-term metabolic trajectories or sustained vascular remodeling, and the study may have had limited statistical power to detect smaller between-group effects, particularly for secondary outcomes and subgroup analyses. Second, the community-based implementation, although enhancing ecological validity, may have introduced variations in dietary intake, physical activity, and adherence that were not fully controlled. Repeated dietary recalls were not systematically collected during the 12-wk intervention; therefore, changes in total energy and macronutrient intake could not be quantified or adjusted for. Although the prescribed cookie dose could be compared with a 2000-kcal reference diet, its contribution to participants’ actual habitual intake could not be determined, limiting our ability to separate the effects of the study cookies from concurrent dietary changes. Future studies should incorporate repeated 24-h dietary recalls or other validated dietary assessment methods to characterize background dietary intake and control for dietary confounding. Third, biomarker assessment was limited to selected vascular and inflammatory indicators, and the single geographic and cultural setting may restrict generalizability. In addition, all study groups received the same standardized FUNCARE nurse-led coaching, and no no-FUNCARE control group was included. Consequently, the independent effect of the nurse-led component cannot be determined; the randomized comparisons primarily estimate the incremental effects of different concentrations of *G. procumbens*–enriched cookies within a common FUNCARE framework. Future multicenter studies with longer follow-up, broader biomarker assessment, and designs capable of separating the effects of functional nutrition from nurse-led behavioral support are warranted.

A further methodological limitation concerns the scope of phytochemical characterization. Comparative GC-MS profiling of the pure *G. procumbens* extract, placebo cookie, 5%, 10%, and 20% cookie formulations, together with targeted HPLC analysis of selected nonvolatile phytochemical markers, improved chemical characterization and source attribution but did not provide comprehensive profiling of the full phytochemical spectrum. Although all intervention formulations were subjected to comparative phytochemical characterization, the analyses were limited to selected volatile, semivolatile, and targeted nonvolatile phytochemical markers and therefore cannot establish the contribution of individual constituents to the observed clinical effects. Future studies should incorporate comprehensive untargeted LC-MS/MS profiling, pharmacokinetic assessment of relevant bioactive compounds, and predefined batch-to-batch phytochemical standardization.

In conclusion, this randomized controlled trial showed that, within a standardized nurse-led FUNCARE framework, higher concentrations of *G. procumbens*–enriched cookies were associated with lower HbA1c and HOMA-IR, particularly at the 10% and 20% concentrations, whereas no significant treatment-group effect was observed for FPG; selected vascular biomarkers also differed compared with placebo. The intervention was feasible, acceptable, and well tolerated in a community-based setting. Because all groups received the same FUNCARE coaching, the independent effect of the nurse-led component cannot be determined. These findings support further evaluation of *G. procumbens*–based functional nutrition delivered within structured nurse-led diabetes care, including future factorial designs capable of separating the effects of functional nutrition from those of nurse-led behavioral support. These findings support the potential of *G. procumbens*–enriched functional foods as an adjunct to standardized nurse-led behavioral support for improving selected, rather than all, metabolic and vascular outcomes. High adherence and acceptability, together with the absence of serious adverse events, further support the feasibility of delivering this combined approach in community health settings. Further studies are warranted to confirm the clinical relevance, long-term safety, and sustainability of these effects and to independently evaluate the contribution of the nurse-led FUNCARE component.

## Author contributions

The authors’ responsibilities were as follows – AS, NJ: designed the research; AS, NJ, WFD, PCS: conducted the research; WFD, PCS: provided essential materials and technical support related to the preparation and characterization of *Gynura procumbens*–enriched cookies; AS, NJ: analyzed data and performed statistical analysis; AS, NJ: wrote the paper; AS: had primary responsibility for final content; and all authors: read and approved the final manuscript.

## Data availability

The datasets generated and/or analyzed during this study are available from the corresponding author upon reasonable request, subject to applicable ethical and institutional requirements.

## Declaration of generative AI and AI-assisted technologies in the writing process

During the preparation of this work, the authors used ChatGPT, developed by OpenAI, only to assist with language editing. The tool was not used for data collection, data analysis, interpretation of findings, or generation of scientific conclusions. After using this tool, the authors reviewed and edited the content as needed and take full responsibility for the content of the publication.

## Funding

This work was supported by the Ministry of Higher Education, Science, and Technology, Republic of Indonesia, through the Kemdiktisaintek Research Grant Program 2025 under the Applied Research–Model Output scheme (Contract No. 02209/UN4.22/PT.01.03/2025). The funder had no role in the study design, data collection, data management, data analysis, interpretation of findings, writing of the manuscript, or the decision to submit the manuscript for publication.

## Conflicts of interest

The authors report no conflicts of interest.
