# Supplementary material for: Nurse-Led Functional Nutrition Care Model (FUNCARE): Integrating Biologic and Behavioral Nursing Approaches to Improve Glycemic Outcomes in Type 2 Diabetes
Source: Curr Dev Nutr. 2026 Oct 17;10(11):109555. doi: 10.1016/j.cdnut.2026.109555 (PMC13769085; doi:10.1016/j.cdnut.2026.109555)
Supplement: Supplementary file 1 [file mmc1.docx]

Supporting Information

Nurse-Led Functional Nutrition Care Model (FUNCARE): Integrating Biological and Behavioral Nursing Approaches to Improve Glycemic Outcomes in Type 2 Diabetes

First author: Andina Setyawati

Supplementary Table S1. Vascular Function, MMP-9, TIMP-1, and MMP-9/TIMP-1 Ratio Before and After the Intervention

| **Outcome** | **Group** | **Pre (Mean ± SD)** | **Post (Mean ± SD)** | **Change (%)** | **p-value** |
| --- | --- | --- | --- | --- | --- |
| ABI | Placebo | 0.88 ± 0.06 | 0.89 ± 0.05 | +1.1 | 0.39 |
|  | 5% G. procumbens | 0.90 ± 0.12 | 0.92 ± 0.16 | +2.2 | 0.12 |
|  | 10% G. procumbens | 0.89 ± 0.08 | 0.93 ± 0.17 | +4.5 | 0.041* |
|  | 20% G. procumbens | 0.87 ± 0.13 | 0.94 ± 0.09 | +8.0 | 0.006** |
| MMP-9 (ng/mL) | Placebo | 322.4 ± 61.2 | 318.5 ± 57.2 | -1.2 | 0.58 |
|  | 5% G. procumbens | 335.8 ± 68.9 | 320.2 ± 62.7 | -4.6 | 0.21* |
|  | 10% G. procumbens | 341.2 ± 72.3 | 309.5 ± 59.1 | -9.3 | 0.033* |
|  | 20% G. procumbens | 338.3 ± 66.4 | 286.1 ± 55.8 | -15.4 | 0.009** |
| TIMP-1 (ng/mL) | Placebo | 169.4 ± 29.9 | 168.8 ± 27.3 | -0.4 | 0.77 |
|  | 5% G. procumbens | 172.3 ± 31.1 | 175.8 ± 29.2 | +2.0 | 0.44 |
|  | 10% G. procumbens | 170.8 ± 33.2 | 178.2 ± 33.3 | +4.3 | 0.18 |
|  | 20% G. procumbens | 172.4 ± 28.4 | 187.7 ± 31.4 | +8.9 | 0.030* |
| MMP-9 / TIMP-1 Ratio | Placebo | 1.90 ± 0.42 | 1.89 ± 0.40 | -0.5 | 0.63 |
|  | 5% G. procumbens | 1.95 ± 0.47 | 1.83 ± 0.33 | -6.2 | 0.14 |
|  | 10% G. procumbens | 2.01 ± 0.49 | 1.74 ± 0.38 | -13.4 | 0.028* |
|  | 20% G. procumbens | 1.98 ± 0.45 | 1.54 ± 0.36 | -22.2 | 0.005** |

Values are presented as mean ± SD. ABI, ankle-brachial index; MMP-9, matrix metalloproteinase-9; TIMP-1, tissue inhibitor of metalloproteinase-1. Change (%) was calculated as [(post-intervention value − pre-intervention value) / pre-intervention value] × 100. Within-group differences between pre- and post-intervention values were analyzed using paired t-tests or Wilcoxon signed-rank tests, as appropriate. *p < 0.05, **p < 0.01.

Supplementary Table S2. Preliminary sensory evaluation of G. procumbens cookie formulations prior to the clinical trial.

| **Formulation** | **Appearance** | **Aroma** | **Taste** | **Texture** | **Overall acceptability** |
| --- | --- | --- | --- | --- | --- |
| 5% | 8.1 ± 0.7 | 7.9 ± 0.8 | 8.0 ± 0.7 | 8.1 ± 0.6 | 8.0 ± 0.6 |
| 10% | 7.8 ± 0.8 | 7.7 ± 0.8 | 7.7 ± 0.9 | 7.8 ± 0.8 | 7.8 ± 0.7 |
| 20% | 7.3 ± 0.9 | 7.2 ± 0.9 | 7.0 ± 1.0 | 7.2 ± 0.9 | 7.2 ± 0.8 |
| 25% | 6.0 ± 1.1 | 5.9 ± 1.2 | 5.3 ± 1.3 | 5.8 ± 1.2 | 5.6 ± 1.1 |

Supplementary Table S3. Laboratory Safety Parameters and Adverse Events During the 12-Week Intervention

| **Safety outcome** | **Time point** | **Placebo** | **5% *G. procumbens*** | **10% *G. procumbens*** | **20% *G. procumbens*** | **p-value** |
| --- | --- | --- | --- | --- | --- | --- |
| **Laboratory safety parameters** |  | **n = 60** | **n = 61** | **n = 61** | **n = 61** |  |
| AST (U/L) | Baseline | 24.8 ± 6.1 | 25.1 ± 6.4 | 24.6 ± 5.9 | 25.0 ± 6.2 | — |
|  | Week 12 | 24.5 ± 5.8 | 24.9 ± 6.0 | 24.7 ± 5.7 | 25.3 ± 6.1 | 0.81 |
| ALT (U/L) | Baseline | 26.4 ± 7.2 | 26.8 ± 7.5 | 27.1 ± 7.0 | 26.9 ± 7.4 | — |
|  | Week 12 | 26.1 ± 6.9 | 26.6 ± 7.1 | 26.8 ± 6.8 | 27.2 ± 7.0 | 0.76 |
| Serum creatinine (mg/dL) | Baseline | 0.83 ± 0.16 | 0.82 ± 0.15 | 0.84 ± 0.17 | 0.83 ± 0.16 | — |
|  | Week 12 | 0.82 ± 0.15 | 0.83 ± 0.16 | 0.83 ± 0.16 | 0.84 ± 0.17 | 0.88 |
| eGFR (mL/min/1.73 m²) | Baseline | 89.6 ± 11.8 | 90.4 ± 12.1 | 89.1 ± 11.5 | 90.0 ± 11.9 | — |
|  | Week 12 | 90.1 ± 11.5 | 89.8 ± 11.8 | 89.6 ± 11.6 | 89.3 ± 12.0 | 0.91 |
| Clinically significant hepatic abnormality, n (%) | Week 12 | 0 (0) | 0 (0) | 0 (0) | 0 (0) | — |
| Clinically significant renal abnormality, n (%) | Week 12 | 0 (0) | 0 (0) | 0 (0) | 0 (0) | — |
| **Adverse events** |  | **n = 61** | **n = 61** | **n = 62** | **n = 62** |  |
| Any adverse event, n (%) | During follow-up | 2 (3.3) | 4 (6.6) | 6 (9.7) | 8 (12.9) | — |
| Mild gastrointestinal discomfort, n (%) | During follow-up | 2 (3.3) | 4 (6.6) | 6 (9.7) | 8 (12.9) | — |
| Serious adverse event, n (%) | During follow-up | 0 (0) | 0 (0) | 0 (0) | 0 (0) | — |
| Adverse event requiring medical treatment, n (%) | During follow-up | 0 (0) | 0 (0) | 0 (0) | 0 (0) | — |
| Intervention discontinued because of adverse event, n (%) | During follow-up | 0 (0) | 0 (0) | 0 (0) | 0 (0) | — |

Values are presented as mean ± standard deviation (SD) or n (%), as appropriate. Laboratory safety parameters were evaluated at baseline and week 12 among participants with paired measurements. Between-group comparisons of week-12 laboratory values were performed using ANCOVA adjusted for the corresponding baseline value. Adverse events were summarized descriptively in the safety population, defined as all randomized participants who consumed at least one study cookie. Gastrointestinal symptoms were mild and transient and resolved without specific treatment. AST, aspartate aminotransferase; ALT, alanine aminotransferase; eGFR, estimated glomerular filtration rate.

Supplementary Table S4. Nutritional Composition of the Study Cookies per Serving and Daily Prescribed Intake

| **Nutritional parameter** | **Placebo (0% GP)** | **5% GP** | **10% GP** | **20% GP** |
| --- | --- | --- | --- | --- |
| **Serving size, g** | 25 | 25 | 25 | 25 |
| **Prescribed intake, cookies/day** | 2 | 2 | 2 | 2 |
| **Total daily amount, g/day** | 50 | 50 | 50 | 50 |
| **Per 25-g cookie** |  |  |  |  |
| Energy, kcal | 108 | 107 | 107 | 106 |
| Carbohydrate, g | 15.4 | 15.2 | 15.0 | 14.7 |
| Protein, g | 2.1 | 2.1 | 2.2 | 2.2 |
| Total fat, g | 4.3 | 4.3 | 4.3 | 4.3 |
| Dietary fiber, g | 1.0 | 1.2 | 1.4 | 1.8 |
| **Daily intake from two cookies (50 g/day)** |  |  |  |  |
| Energy, kcal/day | 216 | 214 | 214 | 212 |
| Carbohydrate, g/day | 30.8 | 30.4 | 30.0 | 29.4 |
| Protein, g/day | 4.2 | 4.2 | 4.4 | 4.4 |
| Total fat, g/day | 8.6 | 8.6 | 8.6 | 8.6 |
| Dietary fiber, g/day | 2.0 | 2.4 | 2.8 | 3.6 |
| **Energy contribution to a 2,000-kcal reference diet, %** | 10.8 | 10.7 | 10.7 | 10.6 |

Values represent the nutritional composition of the standardized cookie formulations per 25-g serving and the prescribed daily intake of two cookies (50 g/day). The four formulations were designed to be approximately isocaloric, with the primary formulation difference being the concentration of G. procumbens extract. The percentage energy contribution was calculated using a 2,000-kcal/day reference diet and should not be interpreted as the proportion of participants’ actual daily energy intake. Repeated quantitative dietary recalls were not systematically collected during the intervention; therefore, the contribution of the study cookies to individual participants’ total habitual energy and macronutrient intake could not be determined.

Supplementary Table S5. Relative GC–MS peak areas of selected volatile and semi-volatile compounds in pure Gynura procumbens extract, placebo cookie, and 5%, 10%, and 20% G. procumbens cookie formulations.

| **Compound** | **RT (min)** | **Pure extract (%)** | **Placebo (%)** | **5% cookie (%)** | **10% cookie (%)** | **20% cookie (%)** |
| --- | --- | --- | --- | --- | --- | --- |
| Phytol | 11.2 | 3.8 | ND | **0.8** | **1.6** | 2.5 |
| Neophytadiene | 15.6 | 2.6 | ND | **0.5** | **1.0** | 1.7 |
| 2,4-di-*tert*-butylphenol | 19.8 | 1.4 | 0.3 | **0.5** | **0.8** | 1.1 |
| Hexadecanoic acid methyl ester | 28.4 | 1.6 | 5.8 | **5.9** | **6.1** | 6.4 |
| Linoleic acid derivative | 32.7 | 2.1 | 8.7 | **8.8** | **9.0** | 9.3 |
| Oleic acid derivative | 37.6 | 0.9 | 12.4 | **12.5** | **12.7** | 12.9 |

**Supplementary Figure S1.** Comparative GC-MS chromatograms of pure Gynura procumbens extract, placebo cookie, and 5%, 10%, and 20% G. procumbens-enriched cookie formulations. (A) Pure G. procumbens extract; (B) placebo cookie; (C) 5% G. procumbens cookie; (D) 10% G. procumbens cookie; and (E) 20% G. procumbens cookie. Selected volatile and semi-volatile compounds are indicated according to their retention times. ND, not detected.
